# Craniofacial Ciliopathies Reveal Specific Requirements for GLI Proteins during Development of the Facial Midline

**DOI:** 10.1371/journal.pgen.1006351

**Published:** 2016-11-01

**Authors:** Ching-Fang Chang, Ya-Ting Chang, Grethel Millington, Samantha A. Brugmann

**Affiliations:** Division of Plastic Surgery, Department of Surgery and Division of Developmental Biology, Department of Pediatrics, Cincinnati Children’s Hospital Medical Center, Cincinnati; University of Oxford, UNITED KINGDOM

## Abstract

Ciliopathies represent a broad class of disorders that affect multiple organ systems. The craniofacial complex is among those most severely affected when primary cilia are not functional. We previously reported that loss of primary cilia on cranial neural crest cells, via a conditional knockout of the intraflagellar transport protein KIF3a, resulted in midfacial widening due to a gain of Hedgehog (HH) activity. Here, we examine the molecular mechanism of how a loss of primary cilia can produce facial phenotypes associated with a gain of HH function. We show that loss of intraflagellar transport proteins (KIF3a or IFT88) caused aberrant GLI processing such that the amount of GLI3FL and GLI2FL was increased, thus skewing the ratio of GLIFL to GLIR in favor of the FL isoform. Genetic addition of GLI3R partially rescued the ciliopathic midfacial widening. Interestingly, despite several previous studies suggesting midfacial development relies heavily on GLI3R activity, the conditional loss of GLI3 alone did not reproduce the ciliopathic phenotype. Only the combined loss of both GLI2 and GLI3 was able to phenocopy the ciliopathic midfacial appearance. Our findings suggest that ciliopathic facial phenotypes are generated via loss of both GLI3R and GLI2R and that this pathology occurs via a de-repression mechanism. Furthermore, these studies suggest a novel role for GLI2R in craniofacial development.

## Author Summary

Primary cilia are ubiquitous organelles that serve to transduce molecular signals within a cell. Loss of functional primary cilia results in a disease class called ciliopathies. Ciliopathies have a broad range of phenotypes; however, severe facial anomalies are commonly associated with this disease class. The facial midline is particularly sensitive to loss of primary cilia, frequently undergoing a significant widening. This phenotype is similar to that which occurs when there are gain-of-function defects in the Sonic Hedgehog pathway. This manuscript addresses the molecular basis for midfacial widening in ciliopathies. Importantly, we determine mechanisms to both rescue and phenocopy the ciliopathic midfacial phenotype. In sum, this work provides novel insight into the molecular mechanisms of midfacial patterning and the extent to which loss of cilia impact that process.

## Introduction

Midfacial disorders encompass a spectrum of conditions that affect the development of the facial midline. The full spectrum of medial craniofacial dysplasias range from conditions that exhibit tissue deficiencies or agenesis (hypotelorism, cyclopia) to those that exhibit tissue excess or duplication (hypertelorism, frontonasal dysplasia). The etiology of these conditions are heterogeneous; however, there has been an established linkage between activity of the Hedgehog (HH) pathway and midfacial growth [1–7]. Reduced levels of HH activity are associated with a collapse of midfacial tissues [2, 8, 9], whereas increased levels of HH activity are associated with a widening or duplication of midfacial tissues [1, 7, 10, 11]. Although this correlation between the HH pathway and midfacial disorders is well established, the cellular and molecular mechanisms by which these disorders occur remain nebulous.

GLIs are the major transcriptional effectors of the HH pathway. In vertebrates, the GLI family of proteins consists of three members (GLI1-3). GLI2 and GLI3 act as bifunctional transcription factors that contain an N-terminal repression domain, as well as a C-terminal transcriptional activation domain. Both proteins can be converted from full-length (GLIFL) transcriptional activators (GLIA) into truncated repressors (GLIR) through regulated proteolytic processing [12, 13]. GLI2 acts as the primary transcriptional activator of the HH pathway [14], yet it has been reported to contribute to some repressor activity [15–17]. GLI3 primarily functions as a transcriptional repressor of the HH pathway [18–21], although it has been shown to exert weak activator activity as well [22–24]. There is evidence of GLI2 and GLI3 having distinct, partially redundant roles during development [25], yet it is unclear if and how they compensate for one another during craniofacial development. The ratio of GLIA to GLIR is believed to dictate the net activity of the HH pathway [26, 27]; however, the exact mechanism by which the graded intracellular activity of GLIA and GLIR is generated remains unknown and is the subject of several ongoing studies.

Recently, several groups have contributed to piecing together a potential primary cilia-dependent mechanism for processing of a HH signal (reviewed in [28–30]). Integration of data from these studies allows for the following hypothesized mechanism. Prior to processing, GLI proteins associate with Suppressor of Fused (SUFU), a conserved protein known to regulate the activity of GLI transcription factors via modulating GLI processing, stabilization and subcellular localization [31–34]. In the presence of a HH signal, Smoothened (SMO) is translocated to the cilium [35] and the SUFU-GLIFL complex traffics through the cilium [36]. Activated ciliary SMO then works through KIF7 to promote the dissociation of the inhibitory SUFU-GLIFL complex [33, 34, 37]. Unbound GLIFL is then processed into an activator and moves to the nucleus to promote expression of downstream targets. In the absence of the HH ligand, SMO is not translocated into the cilium and thus cannot antagonize SUFU. SUFU remains in complex with GLI; GLI is proteolytically processed into GLIR and the SUFU-GLIR complex moves to the nucleus where it recruits the Sap18-Sin3 co-repressor complex to repress GLI target genes [38–41].

Loss of functional primary cilia results in a wide range of disorders called ciliopathies. 30% of ciliopathies can be primarily defined by their craniofacial phenotypes. Furthermore, 70% of craniofacial ciliopathies have midfacial defects, reminiscent of those with HH or GLI mutations [42]. To understand how ciliary defects affect GLI-mediated HH pathway activity during midfacial patterning, we conditionally knocked out intraflagellar transport (IFT) proteins in cranial neural crest cells (NCCs) and examined GLI isoform expression and function. Taken together, our data add mechanistic insights into how the cilium and GLI proteins function during midfacial development.

## Results

### Conditional loss of anterograde intraflagellar transport proteins in NCCs produces midfacial defects

**Fig 1:**
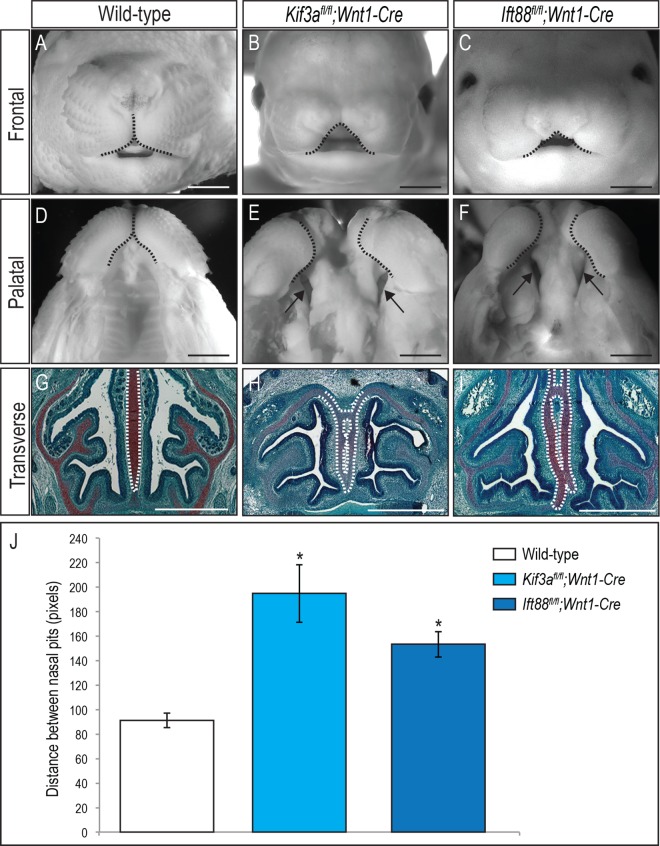
Loss of anterograde intraflagellar transport proteins in NCCs results in severe midfacial widening. (A-C) Frontal view, (D-F) palatal view and (G-I) Safranin-O staining of transverse sections from e15.5 (A, D, G) wild-type, (B, E, H) *Kif3a*^*fl/fl*^*;Wnt1-Cre* and (C, F, I) *Ift88*^*fl/fl*^*;Wnt1-Cre* heads. *Kif3a*^*fl/fl*^*;Wnt1-Cre* and *Ift88*^*fl/fl*^*;Wnt1-Cre* have severe facial widening (B, C; dotted black lines), bilateral cleft of the secondary palate (E, F; black arrows) and duplication of the nasal septum (H, I; dotted white lines). (J) Quantitative measurements of the distance between nasal pits on e13.5 embryos show midfacial widening in both *Kif3a*^*fl/fl*^*;Wnt1-Cre* (n = 8) and *Ift88*^*fl/fl*^*;Wnt1-Cre* (n = 3) is significant, relative to wild-type embryos (n = 12). Statistical analysis was performed by student *t*-test (*P<0.05). Scale bars = 1000 μm.

Our previous work showed that the conditional loss of *Kif3a* in NCCs (*Kif3a*^*fl/fl*^*;Wnt1-Cre*) generated severe midfacial defects, including midfacial widening, duplicated nasal septum, agenesis of the corpus callosum and aglossia [7]. To determine if the midfacial phenotypes were *Kif3a*-specific, or if the loss of other anterograde intraflagellar transport proteins produced this phenotype, we generated *Ift88*^*fl/fl*^*;Wnt1-Cre* embryos and compared the midfacial phenotypes to that of *Kif3a*^*fl/fl*^*;Wnt1-Cre* embryos. *Ift88*^*fl/fl*^*;Wnt1-Cre* embryos had a striking resemblance to *Kif3a*^*fl/fl*^*;Wnt1-Cre* embryos (Fig 1, Tables 1 and 2). Relative to wild-type embryos, both *Kif3a*^*fl/fl*^*;Wnt1-Cre* (n = 28) and *Ift88*^*fl/fl*^*;Wnt1-Cre* (n = 26) exhibited significant midfacial widening, as determined by internasal width (Fig 1A–1C; 1J). Furthermore, both mutants exhibited a bilateral cleft secondary palate (Fig 1D–1F). We had previously determined that the facial skeleton of *Kif3a*^*fl/fl*^*;Wnt1-Cre* mutants was highly dysmorphic [7]. Specifically, there was a duplication of the nasal septum underlying the severe midfacial widening. Safranin-O staining revealed that similar to *Kif3a*^*fl/fl*^*;Wnt1-Cre* embryos, *Ift88*^*fl/fl*^*;Wnt1-Cre* embryos also had a duplicated nasal septum (Fig 1G–1I, n = 2). This striking similarity between the midfacial phenotypes of *Kif3a*^*fl/fl*^*;Wnt1-Cre* and *Ift88*^*fl/fl*^*;Wnt1-Cre* mutants suggested that the underlying mechanisms for midfacial widening were not *Kif3a*-specific, but rather due to a ciliary process in which anterograde IFT proteins participated. Based on data linking midfacial development and primary cilia with the HH pathway, we next examined if GLI post-translational processing was affected in the frontonasal prominence (FNP) of *Kif3a*^*fl/fl*^*;Wnt1-Cre* and *Ift88*^*fl/fl*^*;Wnt1-Cre* embryos.

**Table 1:** Table of genotype and phenotype frequency for wild-type, *Kif3a*^*fl/fl*^*;Wnt1-Cre and Kif3a*^*fl/fl*^*;Wnt1-Cre;Gli3*^*Δ699/+*^.

			Facial phenotype analyzed [n (%)]^‡^		
Genotype	Stage	n (%)^*^	Frontal widening	Bilateral cleft	Duplicated nasal septum
Control	e11.5	117 (73.12%)	0 (0%)	NA	NA
*Kif3a*^*fl/fl*^*;Wnt1Cre*	e11.5	22 (13.75%)	22 (100%)	NA	NA
*Kif3a*^*fl/fl*^*;Wnt1-Cre;Gli3*^*Δ699+/-*^	e11.5	21 (13.13%)	0 (0%)	NA	NA
Control	e14.5	12 (54.55%)	0 (0%)	0 (0%)	0 (0%)
*Kif3a*^*fl/fl*^*;Wnt1-Cre*	e14.5	6 (27.27%)	6 (100%)	6(100%)	6 (100%)
*Kif3a*^*fl/fl*^*;Wnt1-Cre; Gli3*^*Δ699+/-*^	e14.5	4 (18.18%)	0 (0%)	0 (0%)	0 (0%)

Genotypes of control including *Kif3a*^*fl/fl*^, *Kif3a*^*fl/+*^, *Kif3a*^*+/+*^, *Kif3a*^*fl/+*^*;Wnt1Cre*, *Kif3a*^*fl/fl*^*;Gli3d699*^*+/-*^, *Kif3a*^*fl/+*^*;Gli3d699*^*+/-*^, *Kif3a*^*fl/+*^*;Wnt1Cre;Gli3d699*^*+/-*^.

^*^n, number of embryos analyzed; %, percentage relative to the number of total embryos (total embryos for e11.5 n = 160; for e14.5 n = 22).

^‡^n, number of embryos presenting phenotypes; %, percentage relative to the number of embryos analyzed.

Frontal widening is defined by a distance between nasal pits greater than 1.5 mm in e11.5 embryos and greater than 1.8 mm e14.5 embryos.

Bilateral clefting is defined by a patent area greater than 0.103mm^2^ in e14.5 embryos.

**Table 2:** Table of genotype and phenotype frequency for wild-type, *Ift88*^*fl/fl*^*;Wnt1-Cre and Ift88*^*fl/fl*^*;Wnt1-Cre;Gli3*^*Δ699/+*^.

			Facial phenotype analyzed [n (%)]^‡^		
Genotype	stage	n (%)^*^	Frontal widening	Bilateral cleft	Duplicated nasal septum
Control	e11.5	134 (75.70%)	0 (0%)	NA	NA
*Ift88*^*fl/fl*^*;Wnt1-Cre*	e11.5	24 (13.56%)	24 (100%)	NA	NA
*Ift88*^*fl/fl*^*;Wnt1-Cre;Gli3*^*Δ699+/-*^	e11.5	19 (10.74%)	(0%)	NA	NA
Control	e14.5	7 (63.64%)	0 (0%)	0 (0%)	0 (0%)
*Ift88*^*fl/fl*^*;Wnt1-Cre*	e14.5	2 (18.18%)	2 (100%)	2(100%)	2 (100%)
*Ift88*^*fl/fl*^*;Wnt1-Cre; Gli3*^*Δ699+/-*^	e14.5	2 (18.18%)	0 (0%)	0 (0%)	0 (0%)

Genotypes of control including *Ift88*^*fl/fl*^, *Ift88*^*fl/+*^, *Ift88*^*+/+*^, *Ift88*^*fl/+*^*;Wnt1Cre*, *Ift88*^*fl/fl*^*;Gli3d699*^*+/-*^, *Ift88*^*fl/+*^*;Gli3d699*^*+/-*^, *Ift88*^*fl/+*^*;Wnt1Cre;Gli3d699*^*+/-*^.

^*^n, number of embryos analyzed; %, percentage relative to the number of total embryos (total embryos for e11.5 n = 177; for e14.5 n = 11).

^‡^n, number of embryos presenting phenotypes; %, percentage relative to the number of embryos analyzed.

Frontal widening is defined by a distance between nasal pits greater than 1.5 mm in e11.5 embryos and greater than 1.8 mm e14.5 embryos.

Bilateral clefting is defined by a patent area greater than 0.130 mm^2^ in e14.5 embryos

### Loss of *Kif3a* and *Ift88* in NCCs disrupts production of GLI isoforms

**Fig 2:**
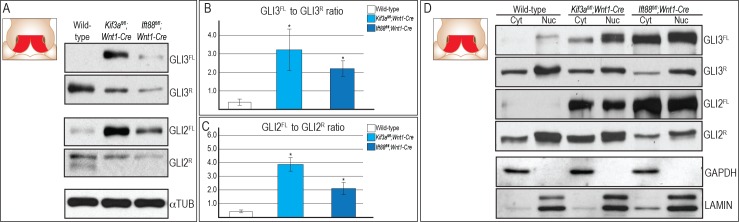
Loss of cilia results in aberrant production of GLI3 and GLI2 isoforms in the FNP. (A) Western blot analysis of GLI3FL, GLI3R, GLI2FL and GLI2R from the FNP of e11.5 embryos (inset schematic, FNP indicated in red). Production of GLI3FL and GLI2FL is increased relative to wild-type embryos, whereas production of GLI3R and GLI2R in both *Kif3a*^*fl/fl*^*;Wnt1-Cre* and *Ift88*^*fl/fl*^*;Wnt1-Cre* is decreased relative to wild-type FNPs. (B, C) Quantitative analysis of Western blot in (A) by ImageJ. The ratios for GLI3FL to GLI3R and GLI2FL to GLI2R are shown. (D) Nuclear fractionation from the FNP of e11.5 embryos shows aberrant levels of GLI3FL, GLI3R, GLI2FL and GLI2R were conserved in the nucleus in both *Kif3a*^*fl/fl*^*;Wnt1-Cre* and *Ift88*^*fl/fl*^*;Wnt1-Cre*. α-Tubulin was used as the loading control for total cell lysate. GAPDH and Lamin A/C were used as the loading control for cytosolic and nuclear fraction, respectively. Inset schematics of facial prominences in A and D indicate FNP (red) was harvested for the experiment.

Primary cilia have previously been implicated in GLI processing [26, 30]. Several ciliary mutants exhibit aberrant production of full-length (FL) and cleaved GLI isoforms, which affects the overall ratio of full-length GLI activator (GLIA) to GLI repressor (GLIR) [26, 43]. To determine if loss of *Kif3a* and *Ift88* impacted the production of GLIFL and GLIR, e11.5 FNPs (area medial to the medial aspect nasal pit and ventral to the dorsal-most aspect of the nasal pit; inset Fig 2A) from each mutant were isolated for Western blot analysis of full-length GLI3 (GLI3FL), cleaved GLI3 (GLI3R), full-length GLI2 (GLI2FL) and cleaved GLI2 (GLI2R) isoforms (Fig 2). The amount of both GLI3FL and GLI2FL isoform was increased in the FNP of both *Kif3a*^*fl/fl*^*;Wnt1-Cre* and *Ift88*^*fl/fl*^*;Wnt1-Cre* mutants (Fig 2A), whereas production of GLI3R and GLI2R in *Kif3a*^*fl/fl*^*;Wnt1-Cre* and *Ift88*^*fl/fl*^*;Wnt1-Cre* embryos was reduced relative to that in the wild-type FNPs (Fig 2A). We next performed densitometry to quantitate how altered production of GLI isoforms affected the overall ratio of GLIFL to GLIR protein. Densitometry analysis determined that there was an approximate 8-fold and 5.5-fold increase of the GLI3FL:GLI3R ratio in the FNPs of *Kif3a*^*fl/fl*^*;Wnt1-Cre* and *Ift88*^*fl/fl*^*;Wnt1-Cre* mutants, respectively (Fig 2B) and an approximate 9-fold and 4.7-fold increase in GLI2FL:GLI2R ratio in the FNPs of *Kif3a*^*fl/fl*^*;Wnt1-Cre* and *Ift88*^*fl/fl*^*;Wnt1-Cre* mutants, respectively (Fig 2C). We speculate that the more significant increase in GLI expression in *Kif3a*^*fl/fl*^:*Wnt1-Cre* mutants relative to *Ift88*^*fl/fl*^*;Wnt1-Cre* mutants stems from the independent function of these proteins within the cilium. The more significant increase in GLI protein expression is in line with the *Kif3a*^*fl/fl*^:*Wnt1-C*re phenotype being slightly more severe than the *Ift88*^*fl/fl*^:*Wnt1-Cre* phenotype. The specificity of the antibodies for GLI2 and GLI3 and the presence of GLI3 and GLI2 full-length and repressor isoforms was confirmed by performing Western blot analysis and noting the absence of bands in *Gli3*^*fl/fl*^*;Wnt1-Cre* and *Gli2*^*fl/fl*^*;Wnt1-Cre* embryos (S1 Fig) and allowing for longer exposure of film (S2 Fig), respectively.

GLI proteins can be degraded in the cytoplasm or shuttled to the nucleus, where they act as transcriptional activators or repressors. To determine if the robust increase in GLI3FL and GLI2FL detected via Western blot was conserved in the nucleus, we performed nuclear fractionation analysis (Fig 2D). Nuclear GLI3FL and GLI2FL were increased in the FNP of both *Kif3a*^*fl/fl*^*;Wnt1-Cre* and *Ift88*^*fl/fl*^*;Wnt1-Cre* embryos, whereas nuclear GLI3R was slightly reduced in the FNP of both *Kif3a*^*fl/fl*^*;Wnt1-Cre* and *Ift88*^*fl/fl*^*;Wnt1-Cre* embryos. Levels of nuclear GLI2R appeared similar between wild-type and *Kif3a*^*fl/fl*^*;Wnt1-Cre* embryos, yet appeared to decrease slightly in *Ift88*^*fl/fl*^*;Wnt1-Cre* embryos. Overall, the loss of cilia in NCCs caused an increase in the amount of nuclear GLI3FL/GLI2FL and a decrease in nuclear GLI3R/GLI2R. Furthermore, this alteration in the production of GLI isoforms resulted in a skewing of the GLIFL to GLIR ratio in favor of the GLIFL isoform.

It is well established that a net gain of HH function produces midfacial widening [1]. The increased GLIFL to GLIR ratio posed two possible molecular mechanisms for producing HH-dependent midfacial widening: (1) a loss of GLIR activity (de-repression) [44], or (2) a gain of GLIA activity. Using genetic and biochemical approaches, we attempted to determine which mechanism was responsible for midfacial widening in ciliopathic mutants.

### Genetic addition of GLI3R in ciliary mutants can partially rescue ciliopathic midfacial phenotypes

**Fig 3:**
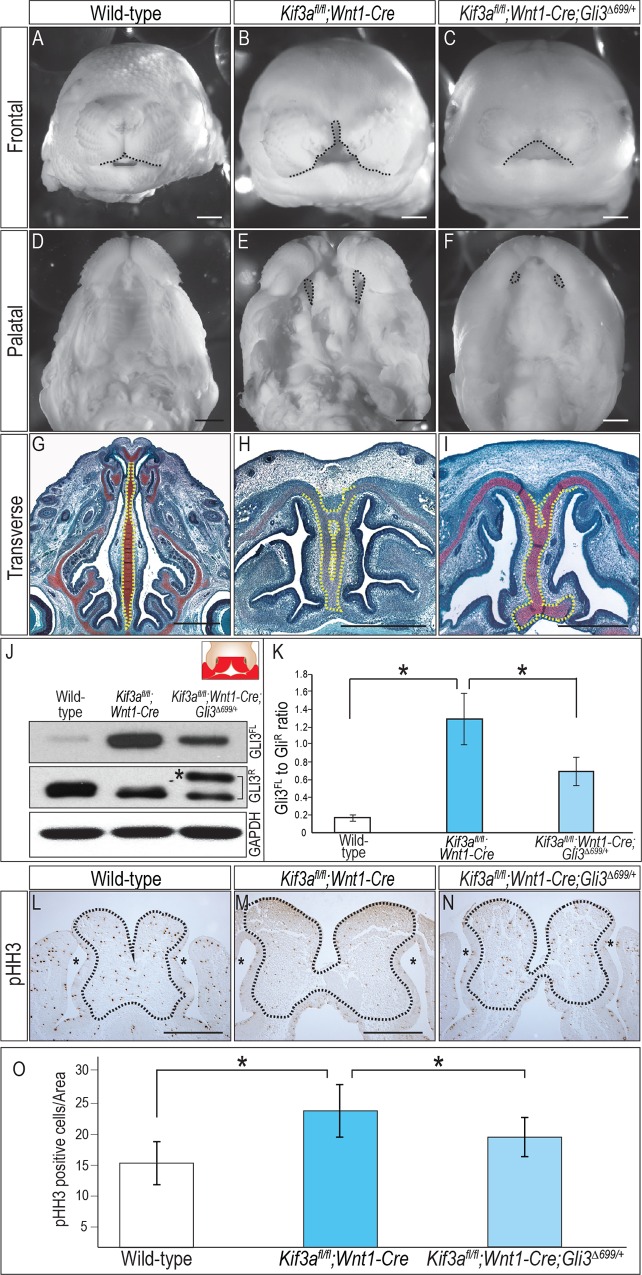
Genetic addition of GLI3R partially rescues *Kif3a*^*fl/fl*^*;Wnt1-Cre* phenotype. (A-C) Frontal view, (D-F) palatal view and (G-I) Safranin-O staining of transverse sections from e15.5 (A, D, G) wild-type, (B, E, H) *Kif3a*^*fl/fl*^*;Wnt1-Cre* and (C, F, I) *Kif3a*^*fl/fl*^*;Wnt1-Cre;Gli3*^*Δ699/+*^ heads. *Kif3a*^*fl/fl*^*;Wnt1-Cre;Gli3*^*Δ699/+*^ showed a significant reduction in the internasal width relative to *Kif3a*^*fl/fl*^*;Wnt1-Cre* (compare B, C; dotted black line) and reduction in patency of bilateral cleft secondary palate (compare E, F; dotted black lines). The duplicated nasal septum of the *Kif3a*^*fl/fl*^*;Wnt1-Cre* was restored to a singular cartilaginous element in *Kif3a*^*fl/fl*^*;Wnt1-Cre;Gli3*^*Δ699/+*^ (compare H and I; dotted yellow lines). (J) Western blot analysis of GLI3 expression in wild-type, *Kif3a*^*fl/fl*^*;Wnt1-Cre* and *Kif3a*^*fl/fl*^*;Wnt1-Cre;Gli3*^*Δ699/+*^ facial prominences. Asterisk denotes expression of GLI3^Δ699R^. GAPDH was used as the loading control. (K) Quantitative analysis of Western blot in (J) by ImageJ (n = 3). GLI3FL to GLI3R ratio is significantly increased in *Kif3a*^*fl/fl*^*;Wnt1-Cre* compared to wild type. However, the ratio is significantly reduced in *Kif3a*^*fl/fl*^*;Wnt1-Cre;Gli3*^*Δ699/+*^ compared to *Kif3a*^*fl/fl*^*;Wnt1-Cre*. Statistical analysis was performed by student *t*-test (*P<0.01), with three separate Western blots. (L-N) Phospho-Histone H3 (pHH3) staining of FNP mesenchyme in e11.5 embryos. (O) Quantitative measurement of pHH3 positive cells in the FNP of wild-type (n = 3; 7 consecutive, 8μm sections), *Kif3a*^*fl/fl*^*;Wnt1-Cre* (n = 3; 7 consecutive 8μm sections) and *Kif3a*^*fl/fl*^*;Wnt1-Cre;Gli3*^*Δ699/+*^ (n = 3; 12 consecutive, 8μm sections) embryos. Statistical analysis was performed by student *t*-test (*P<0.05). Scale bars = 1000 μm. Inset schematic of facial prominences in J indicate FNP, maxillary prominence (MXP) and mandibular prominence (MNP) (red) were harvested for the experiment.

GLI3 predominantly acts as the repressor of the HH pathway (reviewed in [45]), and loss of GLI3R has been linked to midfacial widening [11, 46, 47]. To test if reduced GLI3R is causal for ciliopathic midfacial phenotypes, we genetically increased GLI3R levels with the addition of one allele of *Gli3*^*Δ699*^ [48] into *Kif3a*^*fl/fl*^*;Wnt1-Cre* mutants. *Gli3*^*Δ699*^ encodes a C-terminally truncated GLI3^Δ699^R that mimics the cleaved GLI3R [48]. *Kif3a*^*fl/fl*^*;Wnt1-Cre;Gli3*^*Δ699/+*^ (n = 25) embryos showed a reduction in the internasal width, relative to *Kif3a*^*fl/fl*^*;Wnt1-Cre* mutants (Fig 3A–3C; Table 1). The reduction in midfacial widening was accompanied by a less patent palate (Fig 3D–3F; Table 1). Most notably, the duplicated nasal septum of the *Kif3a*^*fl/fl*^*;Wnt1-Cre* mutants was restored to a singular cartilaginous element in *Kif3a*^*fl/fl*^*;Wnt1-Cre;Gli3*^*Δ699/+*^ embryos (Fig 3G–3I; Table 1). We repeated these rescue experiments by crossing *Gli3*^*Δ699*^ into *Ift88*^*fl/fl*^*;Wnt1-Cre* mutants. Not surprisingly, given the lack of axonemal extension in *Ift88*^*fl/fl*^*;Wnt1-Cre* mutants (S3A and S3B Fig), we observed a similar improvement in the midfacial phenotypes of *Ift88*^*fl/fl*^*;Wnt1-Cre*;*Gli3*^*Δ699/+*^ embryos (n = 21) relative to *Ift88*^*fl/fl*^*;Wnt1-Cre* mutants (S3C–S3E Fig; Table 2). Thus, based on these three phenotypic characteristics, we concluded that genetic addition of GLI3R produced a partial rescue of ciliopathic midfacial widening. We referred to the rescue as partial, because, despite a phenotypic improvement, *Kif3a*^*fl/fl*^*;Wnt1-Cre;Gli3*^*Δ699/+*^ and *Ift88*^*fl/fl*^*;Wnt1-Cre*;*Gli3*^*Δ699/+*^ embryos still exhibited craniofacial anomalies: midfacial widening (compare Fig 3A and 3C) and shorter proximal-distal length along the nasal septum (compare Fig 3G and 3I; S3C and S3E Fig). *Gli3*^*Δ699/+*^ and *Gli3*^*Δ699/∆699*^ embryos did not display any obvious craniofacial defects, despite the reduction or absence of GLI3A (S4 Fig), further supporting the hypothesis that loss of the GLI3R rather than the gain of the GLI3A contributed to the midfacial widening.

To confirm that addition of one *Gli3*^*Δ699*^ allele molecularly restored the amount of GLI3R to wild-type levels, we performed Western blot analysis on e11.5 wild-type, *Kif3a*^*fl/fl*^*;Wnt1-Cre* and *Kif3a*^*fl/fl*^*;Wnt1-Cre;Gli3*^*Δ699/+*^ facial prominences (inset Fig 3J). Introduction of GLI3^Δ699^R (Fig 3J asterisk) increased the total amount of GLI3R in *Kif3a*^*fl/fl*^*;Wnt1-Cre;Gli3*^*Δ699/+*^ embryos to a level comparable to wild-type, and thus restored the ratio of GLI3FL to GLI3R closer to that of wild-type embryos (Fig 3K). Again, these results were similar to what was observed in *Ift88*^*fl/fl*^*;Wnt1-Cre;Gli3*^*Δ699/+*^ facial prominences (S3F Fig). Finally, our previous work suggested that the onset of midfacial widening in *Kif3a*^*fl/fl*^*;Wnt1-Cre* was due to increased midfacial proliferation within the FNP prior to condensation of the nasal septum [7]. Phospho-Histone H3 (pHH3) staining confirmed increased proliferation in the distal FNP of *Kif3a*^*fl/fl*^*;Wnt1-Cre*, relative to wild-type embryos (Fig 3L and 3M). Introduction of the *Gli3*^*Δ699*^ allele significantly reduced pHH3 staining, suggesting that proliferation was restored to that of wild-type levels (Fig 3N and 3O; n = 3). These data, in conjunction with partial rescue of the craniofacial phenotype, suggested that aberrant HH pathway activity, via reduced GLI3R activity, was the molecular basis of ciliopathic midfacial widening.

### GLI3 binding to GLI binding regions is altered in ciliary mutants

**Fig 4:**
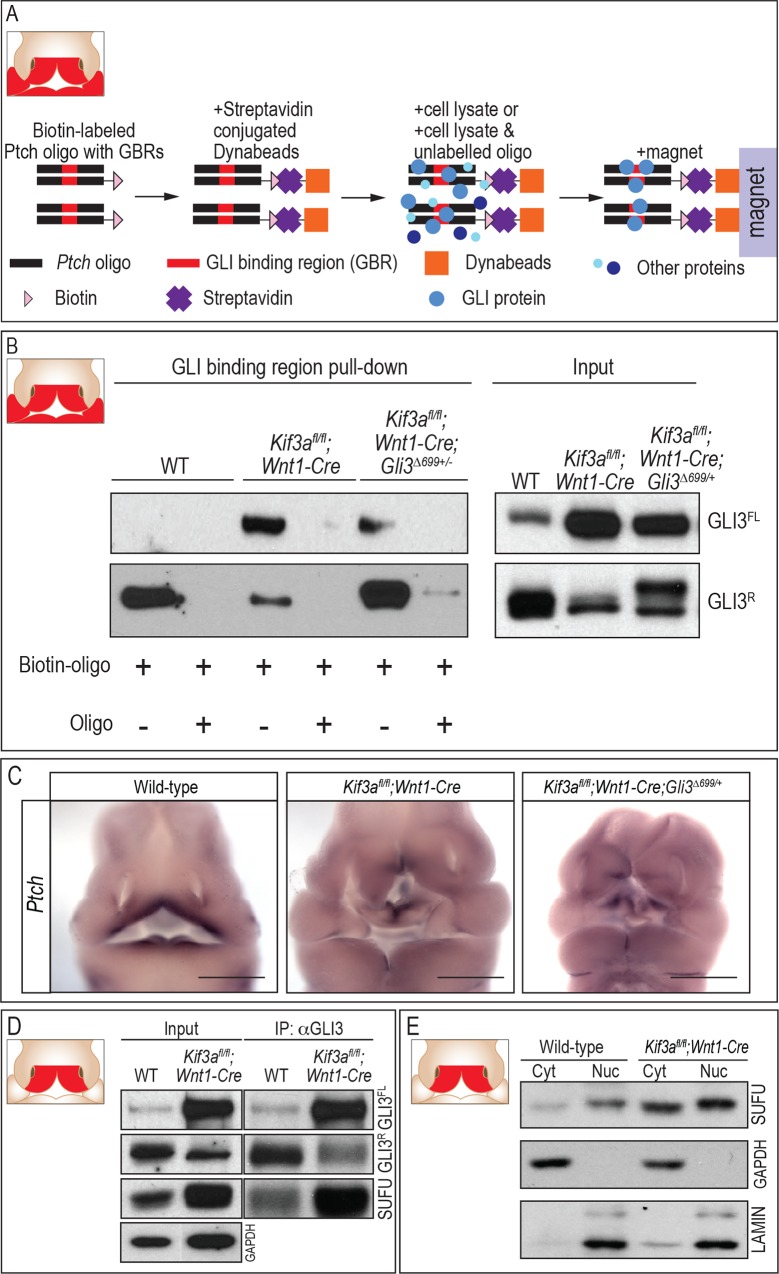
GLI isoforms binding to GLI binding regions is aberrant in ciliary mutants. (A) Schematic diagram of experimental design. (B) Pull-down of GLI3 protein using *Ptch* oligo containing GBR (n = 3). (C) Whole mount *in situ* hybridization of *Ptch* in wild-type, *Kif3a*^*fl/fl*^*;Wnt1-Cre* and *Kif3a*^*fl/fl*^*;Wnt1-Cre;Gli3*^*Δ699/+*^ embryos. (D) SUFU pull down by GLI3 in the cytosolic fraction of FNPs from wild-type and *Kif3a*^*fl/fl*^*;Wnt1-Cre* embryos. GAPDH was used as a loading control. (E) Nuclear fractionation of SUFU in wild-type and *Kif3a*^*fl/fl*^*;Wnt1-Cre* FNPs. Lamin and GAPDH were used as loading control for nuclear and cytosolic fraction, respectively. Scale bars in C = 1000 μm. Inset schematics of facial prominences in A, B, D, E indicate FNP, maxillary prominence (MXP) and mandibular prominence (MNP) were harvested for experiments in A and B, while only the FNP (red) was harvested for experiments in D and E.

GLI proteins act as the key transcription factors for transduction of a HH signal. To do so, GLI proteins bind to consensus GLI binding regions (GBRs) within the regulatory regions of target genes. To determine if GLI3 generated in ciliary mutants was able to recognize and occupy GBRs, we generated a biotin-labeled oligo for the *Patched* (*Ptch*) promoter containing one endogenous GBR (Fig 4A). The *Ptch* oligo was incubated with streptavidin conjugated Dynabeads and then with cell lysate (Fig 4A) from the facial prominences of wild-type, *Kif3a*^*fl/fl*^*;Wnt1-Cre* mutants or *Kif3a*^*fl/fl*^*;Wnt1-Cre;Gli3*^*Δ699/+*^ rescue embryos (inset Fig 4A and 4B). Pull-down and subsequent Western blot experiments revealed that in wild-type facial prominences, GLI3R dominated the binding of GBRs (Fig 4B). We repeated this experiment in *Kif3a*^*fl/fl*^*;Wnt1-Cre* mutant facial prominences and found two interesting results. First, despite lacking proper ciliary-dependent post-translational processing, GLI3 generated in ciliary mutants was still able to recognize and bind to GBRs. Second, the distribution of GLI3FL to GLI3R binding to the synthesized GBRs was altered in the facial prominences of the *Kif3a*^*fl/fl*^*;Wnt1-Cre* mutant relative to wild-type facial prominences. In *Kif3a*^*fl/fl*^*;Wnt1-Cre* mutants GLI3R binding was reduced while GLI3FL binding was increased relative to the wild-type (Fig 4B). We next repeated this experiment with the facial prominences of *Kif3a*^*fl/fl*^*;Wnt1-Cre;Gli3*^*Δ699/+*^ embryos that partially rescued the *Kif3a*^*fl/fl*^*;Wnt1-Cre* midfacial phenotypes. GLI3FL binding to GBRs was reduced and GLI3R binding to GBRs was drastically increased in *Kif3a*^*fl/fl*^*;Wnt1-Cre;Gli3*^*Δ699/+*^ embryos, making the GBR binding profile of the *Kif3a*^*fl/fl*^*;Wnt1-Cre;Gli3*^*Δ699/+*^ more equivalent to wild-type embryos than *Kif3a*^*fl/fl*^*;Wnt1-Cre* mutant embryos (Fig 4B). Thus, *Kif3a*^*fl/fl*^*;Wnt1-Cre;Gli3*^*Δ699/+*^ embryos appeared to rescue the ciliopathic midfacial defects phenotypically, molecularly, and at the level of chromatin binding.

Our GBR pull-down assay was performed *in vitro* with a synthesized *Ptch* oligo containing a GBR. To test if altered levels of GLI isoform binding to regulatory regions could impact target gene expression *in vivo*, we performed *Ptch in situ* hybridization on e11.5 wild-type, *Kif3a*^*fl/fl*^*;Wnt1-Cre* and *Kif3a*^*fl/fl*^*;Wnt1-Cre;Gli3*^*Δ699/+*^ embryos (Fig 4C). As expected, wild-type embryos displayed *Ptch* expression in previously defined areas of the facial prominences. Interestingly, *Ptch* expression was decreased throughout the facial prominences in *Kif3a*^*fl/fl*^*;Wnt1-Cre* embryos (Fig 4C). Furthermore, GLI1 protein expression was not changed in *Kif3a*^*fl/fl*^*;Wnt1-Cre* embryos (S5 Fig). These data suggest that the loss of cilia disrupts the pathway in such manner that expression of the two pathway ‘readouts’ are neither synchronized, nor accurate representations of pathway dynamics, a trend we have observed before with ciliary mutants [49]. The addition of the *Gli3*^*Δ699*^ allele in *Kif3a*^*fl/fl*^*;Wnt1-Cre;Gli3*^*Δ699/+*^ embryos did not restore *Ptch* expression to that of the wild-type (Fig 4C). Thus, despite having increased levels of GLI3FL produced in *Kif3a*^*fl/fl*^*;Wnt1-Cre* mutants (Fig 2) and increased enrichment of GLI3FL on the GBRs of a synthesized oligo, we did not see increased expression of *Ptch in vivo*. These data suggest that the partial rescue of midfacial widening in *Kif3a*^*fl/fl*^*;Wnt1-Cre;Gli3*^*Δ699/+*^ embryos is not via restoration of *Ptch* expression and that increased production of GLI3FL does not translate into increased activator activity in *Kif3a*^*fl/fl*^*;Wnt1-Cre* mutants. We next attempted to determine why GLI3FL generated in *Kif3a*^*fl/fl*^*;Wnt1-Cre* mutants was not functioning as an activator.

### Production and GLI3 binding to SUFU is altered in ciliary mutants

When HH ligand is present, SMO is trafficked into the cilia. Activated SMO, in conjunction with KIF7, dissociates GLIFL from SUFU, allowing GLIFL to function as an activator. Absence of HH ligand or loss of cilia prevents SMO localization to the ciliary axoneme, and thus SMO/KIF7 mediated dissociation of the GLI-SUFU complex is impaired, rendering GLIFL under constitutive inhibition, leading to the loss of GLI activator activity [50]. To determine if excess GLIFL produced in *Kif3a*^*fl/fl*^*;Wnt1-Cre* embryos remained associated with SUFU, we examined the association of SUFU with GLI3 in the developing FNP (inset Fig 4D). In wild-type embryos, low levels of SUFU protein were detected. There was an increase of total SUFU protein in *Kif3a*^*fl/fl*^*;Wnt1-Cre* mutant embryos (Fig 4D, input), as well as an increase in the amount of GLI3-SUFU association (Fig 4D, IP:αGLI3). Furthermore, we performed nuclear fractionation and found levels of nuclear SUFU were increased in the *Kif3a*^*fl/fl*^*;Wnt1-Cre* mutant (Fig 4E). Taken together, these data suggest that despite increased amounts of GLI3FL in *Kif3a*^*fl/fl*^*;Wnt1-Cre* mutant embryos, GLI3FL was rendered inactive, possibly due to a failure to dissociate from SUFU.

### Conditional loss of GLI3 in NCCs does not recapitulate the midfacial phenotype of ciliary mutants

**Fig 5:**
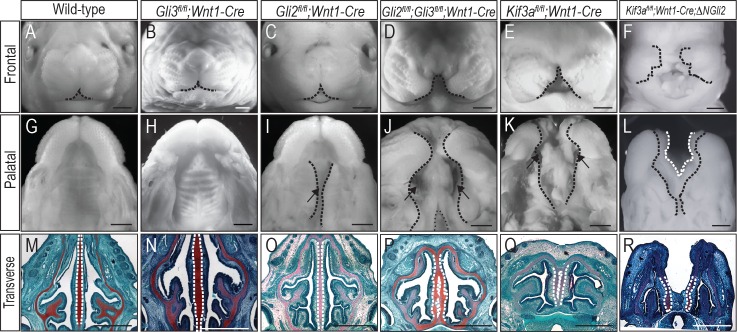
Combined conditional loss of GLI3 and GLI2 phenocopies midfacial phenotypes of ciliary mutants. (A-F) Frontal view, (G-L) palatal view and (M-R) Safranin-O staining on transverse sections of the nasal septum (dotted white lines) from e15.5 (A, G, M) wild-type, (B, H, N) *Gli3*^*fl/fl*^*;Wnt1-Cre* (n = 8), (C, I, O) *Gli2*^*fl/fl*^*;Wnt1-Cre* (n = 3), (D, J, P) *Gli2*^*fl/fl*^*;Gli3*^*fl/fl*^*;Wnt1-Cre* (n = 6), (E, K, Q) *Kif3a*^*fl/fl*^*;Wnt1-Cre* and (F, L, R) *Kif3a*^*fl/fl*^*;Wnt1-Cre;∆NGli2* (n = 5). Scale bars = 1000 μm.

Multiple studies have suggested GLI3 predominantly functions as a repressor, and that GLI3R activity is required for midfacial patterning [11, 46, 47]. Based on these findings and the partial rescue of ciliopathic midfacial phenotypes in *Kif3a*^*fl/fl*^*;Wnt1-Cre;Gli3*^*Δ699/+*^ embryos, we hypothesized that loss of GLI3 in NCCs should recapitulate the ciliopathic midfacial widening observed in *Kif3a*^*fl/fl*^*;Wnt1-Cre* and *Ift88*^*fl/fl*^*;Wnt1-Cre* mutants. To test this hypothesis, we generated *Gli3*^*fl/fl*^*;Wnt1-Cre* embryos. Interestingly, we found that *Gli3*^*fl/fl*^*;Wnt1-Cre* embryos did not phenocopy the midfacial phenotypes of ciliary mutants (Fig 5). *Gli3*^*fl/fl*^*;Wnt1-Cre* embryos did not show midfacial widening (Fig 5A and 5B), a cleft secondary palate (Fig 5G and 5H) or a bifurcated nasal septum (Fig 5M and 5N). The lack of midfacial phenotypes in *Gli3*^*fl/fl*^*;Wnt1-Cre* embryos suggested that perhaps GLI3 was not the only factor driving the midfacial ciliopathic phenotype. Given that GLI2 processing is also disrupted in *Kif3a*^*fl/fl*^*;Wnt1-Cre* and *Ift88*^*fl/fl*^*;Wnt1-Cre* mutants (Fig 2), and that GLI2 null animals have craniofacial defects [25], we hypothesized that GLI2 may also be playing an important role in midfacial patterning.

### Conditional loss of both GLI2 and GLI3 in NCCs recapitulates the midfacial phenotype of ciliary mutants

A precedent exists for GLI proteins compensating for one another in several biological contexts and for GLI2 having repressor activity [16, 17]. To determine if loss of GLI2 function also contributed to the ciliopathic midfacial phenotype, we generated *Gli2*^*fl/fl*^*;Wnt1-Cre* and *Gli2*^*fl/fl*^*;Gli3*^*fl/fl*^*;Wnt1-Cre* mutants and assayed embryos for characteristic midfacial phenotypes of ciliopathic mutants. Similar to *Gli3*^*fl/fl*^*;Wnt1-Cre* embryos, *Gli2*^*fl/fl*^*;Wnt1-Cre* embryos did not show midfacial widening (Fig 5C), but did have a medial cleft of the secondary palate (Fig 5I). Furthermore, although a small split in the most ventral aspect of the nasal septum was detected in *Gli2*^*fl/fl*^*;Wnt1-Cre* embryos (S6 Fig), these embryos did not present with a bifurcated septum similar to that of the *Kif3a*^*fl/fl*^*;Wnt1-Cre* and *Ift88*^*fl/fl*^*;Wnt1-Cre* mutants (Fig 5O). Overall, *Gli2*^*fl/fl*^*;Wnt1-Cre* embryos did not phenocopy *Kif3a*^*fl/fl*^*;Wnt1-Cre* and *Ift88*^*fl/fl*^*;Wnt1-Cre* mutants.

We next tested if conditional loss of both GLI2 and GLI3 in NCCs would recapitulate the midfacial ciliopathic phenotype. *Gli2*^*fl/fl*^*;Gli3*^*fl/fl*^*;Wnt1-Cre* double mutants exhibited facial phenotypes very similar to *Kif3a*^*fl/fl*^*;Wnt1-Cre* and *Ift88*^*fl/fl*^*;Wnt1-Cre* mutants. *Gli2*^*fl/fl*^*;Gli3*^*fl/fl*^*;Wnt1-Cre* embryos exhibited midfacial widening (Fig 5D; Table 3), bilateral clefting of the secondary palate (Fig 5J; Table 3) and a bifurcated nasal septum (Fig 5P; Table 3), giving the *Gli2*^*fl/fl*^*;Gli3*^*fl/fl*^*;Wnt1-Cre* embryos a strikingly similar phenotype to *Kif3a*^*fl/fl*^*;Wnt1-Cre* and *Ift88*^*fl/fl*^*;Wnt1-Cre* embryos (compare Fig 5D, 5E; 5J, 5K; and 5P, 5Q; Table 3). These data, together with our biochemical analysis showing aberrant GLI2 and GLI3 processing (Fig 2), suggest that the midfacial widening in ciliopathic mutants is caused by the combinatorial loss of GLI2R and GLI3R function.

**Table 3:** Table of genotype and phenotype frequency for wild-type, *Gli2*^*fl/fl*^*;Gli3*^*fl/+*^ *Wnt1-Cre*, *Gli2*^*fl/+*^*;Gli3*^*fl/fl*^*;Wnt1-Cre and Gli2*^*fl/fl*^*;Gli3*^*fl/fl*^*;Wnt1-Cre*.

			Facial phenotype analyzed [n (%)]^‡^		
Genotype	stage	n (%)^*^	Frontal widening	Bilateral cleft	Duplicated nasal septum
Control	e14.5	15 (68.18%)	0 (0%)	0 (0%)	0 (0%)
*Gli2*^*fl/fl*^*;Gli3*^*fl/+*^*;Wnt1-Cre*	e14.5	2 (9.09%)	0 (0%)	0 (0%)	0 (0%)
*Gli2*^*fl/+*^*;Gli3*^*fl/fl*^*;Wnt1-Cre*	e14.5	3 (13.64%)	0 (0%)	0 (0%)	0 (0%)
*Gli2*^*fl/fl*^*;Gli3*^*fl/fl*^*;Wnt1-Cre*	e14.5	2 (9.09%)	2 (100%)	2(100%)	2 (100%)
Control	e15.5	21 (63.64%)	0 (0%)	0 (0%)	0 (0%)
*Gli2*^*fl/fl*^*;Gli3*^*fl/+*^*;Wnt1-Cre*	e15.5	3 (9.09%)	0 (0%)	0 (0%)	0 (0%)
*Gli2*^*fl/+*^*;Gli3*^*fl/fl*^*;Wnt1-Cre*	e15.5	5 (15.15%)	0 (0%)	0 (0%)	0 (0%)
*Gli2*^*fl/fl*^*;Gli3*^*fl/fl*^*;Wnt1-Cre*	e15.5	4 (12.12%)	4 (100%)	4(100%)	4 (100%)

Genotypes of control including *Gli2*^*fl/fl*^*;Gli3*^*fl/+*^, *Gli2*^*fl/+*^*;Gli3*^*fl/fl*^, *Gli2*^*fl/+*^*;Gli3*^*fl/+*^, *Gli2*^*fl/+*^*;Gli3*^*fl/+*^*;Wnt1Cre*.

^*^n, number of embryos analyzed; %, percentage relative to the number of total embryos (total embryos for e14.5 n = 22; for e15.5 n = 33).

^‡^n, number of embryos presenting phenotypes; %, percentage relative to the number of embryos analyzed.

Frontal widening is defined by a distance between nasal pits greater than 1.8 mm in e14.5 and e15.5 embryos.

Bilateral clefting is defined by a patent area greater than 0.253mm^2^ in e14.5 and e15.5 embryos

Our data suggested loss of GLIR function was causal to the midfacial widening in the examined ciliary mutants, as we were able to rescue the phenotypes with addition of GLI3R. We next tested if the craniofacial complex in *Kif3a*^*fl/fl*^*;Wnt1-Cre* embryos would be sensitive to altering the GLI ratio (e.g., would increasing the amount of GLIA in these mutants exacerbate midfacial widening). To do so, we utilized *ΔNGli2* mice. *ΔNGli2* encodes a constitutively active form of GLI2 that mimics the action of GLI2A independent of ciliary processing [51]. We first generated *ΔNGli2;Wnt1-Cre* embryos and examined facial phenotypes with one copy of constitutively active GLI2 in NCCs. *ΔNGli2;Wnt1-Cre* embryos did not exhibit a widened midline or duplicated nasal septum (S7A–S7D Fig), presumably due to overriding GLIR activity. However, when we genetically increased the amount of GLI2A on a background with reduced GLIR via generating *Kif3a*^*fl/fl*^*;Wnt1-Cre;∆NGli2* embryos, we observed a significant exacerbation of the internasal width, relative to *Kif3a*^*fl/fl*^*;Wnt1-Cre* mutants (Fig 5F). The exacerbation of midfacial widening was accompanied by a severe midfacial cleft (Fig 5L) and two completely separate nasal septa in *Kif3a*^*fl/fl*^*;Wnt1-Cre;∆NGli2* embryos (Fig 5R). Western blot analysis confirmed the presence of the ΔNGLI2 protein in *Kif3a*^*fl/fl*^*;Wnt1-Cre;∆NGli2* embryos (S7E Fig).

**Fig 6:**
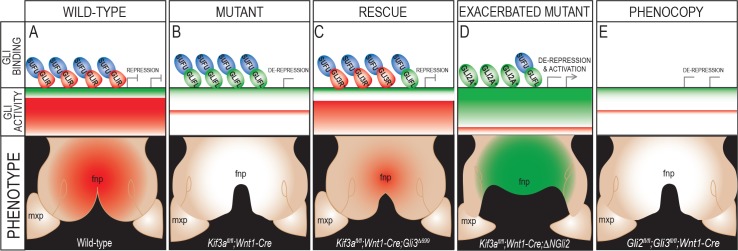
Schematic of hypothesized model for GLI-dependent facial patterning. In (A) wild-type, (B) *Kif3a*^*fl/fl*^*;Wnt1-Cre*, (C) *Kif3a*^*fl/fl*^*;Wnt1-Cre;Gli3*^*Δ699/+*^, (D) *Kif3a^fl/fl^;Wnt1-Cre;ΔNGli2* and (E) *Gli2^fl/fl^; Gli3^fl/fl^; Wnt1-Cre* embryos, GLI binding on GBRs, overall ratio of GLIFL activator versus GLIR activity, and net GLI activity within the developing FNP are indicated. Red indicates GLIR activity, green indicates GLIA activity, white indicates loss of activity. mxp, maxillary prominence. GLI3R* = GLI3^Δ699^R. GLI2A*= ΔNGLI2.

Taken together, these data contribute to the proposal of the following mechanism for midfacial patterning and the observed ciliopathic midfacial phenotypes. In the wild-type FNP, cilia effectively process GLI3FL into GLI3R. GLI3R-SUFU complexes occupy the GBRs within GLI target genes (Fig 6A). A ratio of high GLIR binding relative to GLIFL activator binding at GBRs of target genes is established. Thus, there are high levels of GLIR activity during normal growth of the FNP (Fig 6A). The loss of cilia (in both *Kif3a*^*fl/fl*^*;Wnt1-Cre* and *Ift88*^*fl/fl*^*;Wnt1-Cre* mutants) impairs normal processing of GLIFL and GLIR, resulting in higher levels of GLIFL and lower levels of GLIR, relative to control embryos. Lack of ciliary-localized SMO prevents GLIFL from dissociating from SUFU. Thus, in ciliary mutants there is a reduction in GLIR occupation of GBRs, and the GLI3FL that now occupies the GBR is not functional due to maintained association with SUFU (Fig 6B). This disruption in processing causes a loss of required GLIR activity and a “de-repression” in the FNP. Loss of GLI repression in the FNP then results in midfacial widening, a classic gain of HH phenotype. We further hypothesize that genetic addition of the *Gli3*^*Δ699*^ allele can partially rescue the phenotype via cilia-independent production of a truncated GLI3 protein that functions like GLI3R. This GLI3^Δ699^R is able to compete with GLIFL to occupy the majority of the GBRs within the regulatory regions of target genes (Fig 6C). Binding of GLI3^Δ699^R at GBRs restores the amount of GLIR enrichment and the GLI ratio distribution in the FNP to levels similar to wild-type (Fig 6C). We suggest the rescue is only partial because some amount of GLIFL-SUFU complex still occupies GBRs (supported by data in Fig 4B). Addition of the *ΔNGli2* allele produces GLI2A free from SUFU suppression, and increases the amount of GLI2A at GBRs (Fig 6D). The disruption in cilia-dependent GLI processing combined with addition of functional GLI2A shifts the GLI ratio heavily in favor of the GLIA. The facial phenotype manifests as an exacerbated midfacial widening due to a de-repression combined with increased GLI2A activity in the FNP (Fig 6D). Finally, our hypothesized model also explains how *Gli2*^*fl/fl*^*;Gli3*^*fl/fl*^*;Wnt1-Cre* could phenocopy the *Kif3a*^*fl/fl*^*;Wnt1-Cre* midfacial condition (Fig 6E). Loss of GLI2 and GLI3 would prevent any GLIFL or GLIR from binding to GBRs within the regulatory regions of target genes, thus mimicking the *Kif3a*^*fl/fl*^*;Wnt1-Cre* loss of GLIR binding and increased binding of a non-functional GLIFL-SUFU complex. Loss of both GLI2 and GLI3 in NCCs had the same effect on the GLI production as loss of cilia- loss of the predominant repressor resulting in a de-repression. Loss of GLI repression in the FNP then results in the gain of Hedgehog phenotype of midfacial widening (Fig 6E). Together, these data support the hypothesis that, rather than being dependent upon GLI3R alone, GLI2R also plays a role in midfacial patterning.

## Discussion

It is well established that the HH pathway plays a critical role in midfacial patterning; however, the mechanisms by which HH signaling patterns the face and the role primary cilia play in that process are less clear. Our data suggest that primary cilia play an essential role in midfacial development via the post-translational processing of GLI transcription factors. We report that loss of cilia, via conditional knock-out of anterograde IFT proteins, results in a severely widened facial midline (Fig 1). Western blot analysis showed a significant increase in the amount of nuclear GLI3FL and GLI2FL produced in these mutants (Fig 2), thus skewing the ratio of GLIFL to GLIR in favor of the FL isoform. Genetic addition of GLI3R to the mutant partially rescued the midfacial phenotypes (Fig 3), possibly via re-establishing the proper binding ratio of GLI3FL to GLI3R at GBRs (Fig 4). Although there was an increase in GLIFL production, we suggest the full-length isoform is not active due to maintained association between GLI3FL and SUFU and increased levels of nuclear SUFU in the ciliary mutants. Thus, we support a mechanism of de-repression rather than activation for generating midfacial widening (Fig 4). Despite the ability to rescue the midfacial phenotype with addition of GLI3R, the phenotype did not appear to be solely due to the loss of GLI3R function, as conditional knock-out of GLI3 did not recapitulate the phenotype (Fig 5). The midfacial phenotype could only be recapitulated when both GLI2 and GLI3 were lost in NCCs (Fig 5). Taken together, these data provoke several questions regarding our understanding of midfacial patterning and the role of GLI proteins during craniofacial development.

### Loss of cilia on NCCs results in midfacial widening

Midfacial defects encompass a spectrum of diseases ranging from midline collapse (cyclopia) to midline expansion/duplication (frontonasal dysplasia/diprosopus) [1]. The current understanding of HH-mediated midfacial growth hypothesizes a mechanism by which HH activity in the ventral forebrain directly signals to the adjacent facial ectoderm, inducing a competency in the ectoderm prior to NCC arrival into the FNP. As NCCs migrate in between the forebrain and facial ectoderm, NCCs of the FNP signal to the overlying facial ectoderm establishing a Sonic Hedgehog (SHH)-expressing signaling center in the facial ectoderm coined the Frontonasal Ectodermal Zone (FEZ). The FEZ in turn establishes growth zones within the underlying NCC-derived mesenchyme of the FNP that regulate the size and shape of the midface [52–57]. Gain or loss of SHH activity in either signaling center can cause an expansion or loss of midfacial structures, respectively.

Our studies explore a novel mechanism for the generation of midfacial expansion- a NCC-specific, ciliary-dependent mechanism. The loss of functional primary cilia on NCCs recapitulates the midfacial widening similar to those reported when there is a gain of SHH activity in the forebrain or FEZ (Fig 1; [6]). Several explanations exist to explain how these two different mechanisms can generate similar midfacial phenotypes. First, it is possible that midfacial phenotypes in our conditional ciliary mutants are autonomous to the NCC. Second, it is possible that changes in NCC-derived facial mesenchyme induce changes in the neuroectoderm and FEZ that then contribute to the ciliary midfacial phenotype. There is precedence for NCCs exerting a critical effect on the developing forebrain [58–61], and we have previously observed aberrant morphology in the neuroectoderm of *Kif3a*^*fl/fl*^*;Wnt1-Cre* mutant embryos [7]; however, how those dysmorphologies arise is unknown. Exploring if the establishment and/or maintenance of SHH signaling centers in the brain and face are disrupted in ciliary mutants is a focus of our ongoing work.

Third, the midfacial phenotypes observed in anterograde intraflagellar transport mutants could be due to a combinatorial effect of loss of cilia in both NCCs and neural tissue. Despite being the predominantly used driver to examine NCCs, recombination is also known to occur in the dorsal diencephalon and perhaps other areas of the developing brain in *Wnt1-Cre* animals [62]. Given the significant role cilia have in neural patterning [28], it is possible that the midfacial phenotypes observed in *Kif3a*^*fl/fl*^*;Wnt1-Cre* and *Ift88*^*fl/fl*^*;Wnt1-Cre* embryos are due to defects in the developing brain that then have secondary, compounding effects on NCCs that lack cilia. Understanding the role of primary cilia in transducing signals between NCCs and adjacent tissues during craniofacial development will be important for better understanding the relationship between NCCs, the forebrain and facial ectoderm during craniofacial development.

### Various mechanisms of GLI function exist to pattern developing organ systems

Several studies have established that the GLI proteins function to regulate the output of the HH pathway; however, the mechanisms by which these proteins do so are not fully understood. For example, both GLI2 and GLI3 can function as repressors or activators, and there is precedence for organ systems requiring the function of either a GLIR or GLIA for proper development and patterning [18, 48]. The existence of both repressor and activator isoforms allows for several mechanisms of action including activation, de-repression and ratio sensing to establish the proper amount of activity required for development of an organ system. The range of mechanisms used by GLI proteins is fully evident when examining GLI function in organ systems such as the limb and neural tube. In mouse and chick limb buds, a gradient of GLI3R forms inversely to a SHH source in the ZPA [63, 64] and loss of GLI2 has no effect on digit patterning, whereas loss of GLI3 results in polydactyly [65], a phenotype associated with a gain of HH function [66]. Embryos lacking both SHH and GLI3 also have polydactyly similar to that generated when GLI3 alone is lost, suggesting that limb patterning is due to the suppression of GLI3R function [63, 67]. Interestingly, a separate mechanism exists in the neural tube. Both GLI2 and GLI3 are expressed in the neural tube; however, their expression becomes confined to discrete regions. GLI3 is only expressed in the medial and dorsal regions, whereas GLI2 is expressed throughout the entire dorsal-ventral axis. Loss of function studies support a mechanism in which GLI3R activity is required for patterning the dorsal neural tube, while GLI2A activity is required for patterning the ventral regions of the neural tube [18, 68, 69].

Our study suggests that GLIR activity is particularly important in the craniofacial complex, and that both GLI3R and GLI2R can contribute to this repressive activity. Furthermore, our data support both de-repression and cilia-dependent ratio sensing as essential mechanisms for interpreting the net GLI output in the developing face. In addition to gaining mechanistic insight into how cilia and GLI contribute to the patterning of the craniofacial complex, these data further support the hypothesis that GLI proteins use various mechanisms to exert their function on different organ systems. Our future studies will continue to examine how loss of cilia affect GLI protein function. For example, does loss of cilia impact GLI acetylation, phosphorylation, or sumoylation? Would the loss of these modifications impact the ability of the GLI to exert repressor or activator function? GLI proteins have also been reported to interact with co-regulators [70–72]. Does the loss of ciliary processing affect the ability of GLI to interact with co-activators or co-repressors? Examining GLI processing at multiple levels and understanding how each modification impacts overall protein function is an important challenge that lies ahead.

Although our study centers on the role of primary cilia in GLI processing and subsequent HH pathway activity, there are several pieces of evidence that suggest other pathways may also contribute to midfacial phenotypes observed in ciliopathic mutants. First, primary cilia do not function exclusively to transduce the HH pathway; rather, they are considered a hub for several signaling pathways [73–76]. Thus, it is possible that loss of the cilium also affects the Wnt, TGFβ, Notch and/or PDGF pathways; each of which have previously been implicated in midfacial patterning and craniofacial development [77–80]. Second, despite a marked improvement in midfacial patterning with the addition of the GLI3^Δ699^R, a total rescue was not achieved (Fig 3). We reason this could be due to the fact that we are only ‘rescuing’ the GLI deficit, and not addressing other pathways that may be affected by the loss of functional cilia. Examining how pathways other than the HH pathway are affected in craniofacial ciliopathies is a topic of our future work.

Our data explore both the ciliary- and GLI-dependent molecular mechanisms for the onset of midfacial phenotypes in craniofacial ciliopathies. Further understanding how the cilium processes GLI transcription factors, as well as the distinction between GLI2 and GLI3 and their capacity to compensate for one another is a topic that will undoubtedly be useful for future studies and will perhaps provide avenues for therapeutic intervention when considering treatment for craniofacial ciliopathies.

## Materials and Methods

### Transgenic mice

*Wnt1-Cre* and Gli3^*fl/fl*^ (from Jackson laboratory), *Kif3a*^*fl/fl*^ and *Ift88*^*fl/fl*^ (from Dr. Bradley Yoder, University of Alabama at Birmingham), *Gli2*^*fl/fl*^ (from Dr. Alexandra Joyner, Memorial Sloan-Kettering Cancer Center), *Gli3*^*Δ699*^ (from Dr. Chi-Chung Hui, the Hospital for Sick Kids, Canada), *ΔNGli2* (from Andrzej Dlugosz, University of Michigan) were maintained by Veterinary Services of Cincinnati Children’s Hospital Medical Center with IACUC approval. All transgenic lines were outbred and maintained on a CD1 background.

### Histology and immunostaining

e15.5 embryos were harvested and fixed in Bouin’s solution overnight. For Safranin-O staining, tissue was processed for paraffin embedding and staining procedures were followed as described (http://www.ihcworld.com _protocols/special_stains/safranin_o.htm). For phospho-Histone H3 (pHH3) staining (Ser10, Santa Cruz), e11.5 heads were fixed in 4% PFA, paraffin embedded and cut transversely. Staining was done according to manufacturer’s instruction. pHH3-positive cells in the FNP were counted from multiple consecutive sections (total thickness 60 μm).

### Antibodies

The following antibodies were used per manufactures’ instructions: GLI2 (R&D systems; primary concentration 1:500, secondary concentration 1:5,000), GLI3 (R&D systems primary concentration 1:1,000, secondary concentration 1:5,000), SUFU (H300, Santa Cruz primary concentration 1:2,000, secondary concentration 1:5,000), MYC (9B11, Cell Signaling primary concentration 1:2,000, secondary concentration 1:5,000), GAPDH (FL335, Santa Cruz primary concentration 1:10,000, secondary concentration 1:10,000), LAMIN A/C (H110, Santa Cruz primary concentration 1:2,000, secondary concentration 1:5,000), a-TUBULIN (DM1A, Abcam primary concentration 1:15,000, secondary concentration 1:20,000), PHH3 (JBW301, Millipore primary concentration 1:1,000, secondary concentration 1:1,000), (ARL13b, Protein Tech primary concentration 1:1,000, secondary concentration 1:1,000), gamma-TUBULIN (GTU88, Sigma primary concentration 1:1,000, secondary concentration 1:1,000).

### Western blot, nuclear fractionation and immunoprecipitation

e11.5 embryos were harvested and either the frontonasal prominence (FNP; area medial to nasal pits) alone or the FNP, maxillary (MXP) and mandibular prominence (MNP) combined were dissected out. Whole cell lysate from the frontonasal prominence or combined facial prominences were sonicated in RIPA buffer (50 mM Tris-HCl, pH 7.4, 1% NP-40, 0.25% sodium deoxycholate, 150 mM NaCl, 1 mM EDTA) containing protease (Roche) and phosphatase inhibitors (1 mM PMSF, 1 mM Na_3_VO_4_, 10 mM NaF, 60 mM β-glycerophosphate). For nuclear fractionation, FNPs from multiple e11.5 embryos were freshly isolated and digested by 2 mg/mL Collagenase D (Roche) for 20 minutes at 37°C, with gentle shaking. Cytoplasmic protein was extracted using NE-PER^TM^ reagents (CERI buffer, Thermo Scientific) according to manufacturer’s instruction. The remaining nuclear pellet was re-suspended in CERI buffer and sonicated to obtain nuclear protein. For immunoprecipitation, protein samples were first incubated with antibody for 2 hours at 4°C. Dynabeads were then added and incubated for overnight at 4°C.

GLI3FL to GLI3R ratio was measured by quantitating bands in ImageJ. For the ratio of GLI3FL to GLI3R in *Kif3a*^*fl/fl*^*;Wnt1-Cre;Gli3*^*Δ699/+*^ embryos, the amount of GLI3R used to calculate the ratio included the amount of GLI3^Δ699^R plus endogenous GLI3R.

### GLI-binding region pull-down assay

DNA oligos containing partial sequence of mouse *Ptch1* promoter (-911bps to -970bps) were synthesized with or without Biotin labeled at the 5’ end of the anti-sense strand (sense strand: CGCCCCCCCACCCCCAAGCCTGGGATGCACACACGGGGGTTGCCTACCTGGGTGGTCTCT; anti-sense strand: Biotin-AGAGACCACCCAGGTAGGCAACCCCCGTGTGTGCATC CCAGGCTTGGGGGTGGGGGGGCG, underline indicates GLI binding site). 100 pmole of each sense and anti-sense strand (with or without Biotin labeled) was annealed to dsDNA. The Biotin-dsDNA was then incubated with 30 μL of Streptavidin-coupled Dynabeads (Invitrogen) for 15 minutes at room temperature. DNA-Dynabead complex was incubated with whole cell lysate for two hours at 4°C. GLI proteins pulled down by the DNA/beads complex were detected by Western blot.

## Supporting Information

S1 Fig Expressions of GLI2 and GLI3 isoforms in *Gli2*^*fl/fl*^*;Wnt1-Cre* and *Gli3*^*fl/fl*^*;Wnt1-Cre* facial prominences. (A) Western blotting analysis performed with protein extract from all facial prominences of wild-type, *Gli2*^*fl/+*^*;Wnt1-Cre* and *Gli2*^*fl/fl*^*;Wnt1-Cre* embryos. Both GLI2FL and GLI2R were lost or significantly reduced in *Gli2*^*fl/fl*^*;Wnt1-Cre* mutant, indicating that the *Gli2* gene was efficiently knocked out by Cre activity. (B) Western blotting analysis with all facial prominences of wild-type, *Gli3*^*fl/+*^*;Wnt1-Cre* and *Gli3*^*fl/fl*^*;Wnt1-Cre* embryos. GLI3 protein was also largely eliminated in *Gli3*^*fl/fl*^*;Wnt1-Cre* mutant. Inset schematics of facial prominences in A and B indicates FNP, maxillary prominence (MXP) and mandibular prominence (MNP) (red) were harvested for the experiment.
(JPG)

S2 Fig Longer exposure of Western blot and nuclear analysis. (A) Western blot and (B) nuclear fractionation analysis of GLI3FL, GLI3R, GLI2FL and GLI2R from the FNP of e11.5 embryos with a 2min exposure time. Longer exposure reveals presence of isoforms not observed at shorter exposure time (Fig 2; 30sec).
(JPG)

S3 Fig *GLI3*^*Δ699*^*R* partially rescued *Ift88*^*fl/fl*^*;Wnt1-Cre* midfacial phenotype. (A, B) Immunostaining for the axonemal marker Arl13b (green) and basal body marker γTubulin (red). (A) The axoneme and basal body are present in mesenchyme of the FNP of wild-type embryos. (B) The axoneme is lost in the mesenchyme of the FNP of *Ift88*^*fl/fl*^*;Wnt1-Cre* embryos, but γTubulin staining remains, similar to observations in *Kif3a*^*fl/fl*^*;Wnt1-Cre* embryos. Safranin-O staining of transverse sections from e15.5 (C) wild-type, (D) *Ift88*^*fl/fl*^*;Wnt1-Cre* and (E) *Ift88*^*fl/fl*^*;Wnt1-Cre;Gli3*^*Δ699/+*^ heads. The duplicated nasal septum of the *Ift88*^*fl/fl*^*;Wnt1-Cre* was restored to a singular cartilaginous element in *Ift88*^*fl/fl*^*;Wnt1-Cre;Gli3*^*Δ699/+*^ (compare D and E; dotted white lines). (F) Western Blot for GLI3 isoforms. Expression of GLI3^Δ699^R (asterisk) and reduced expression of GLI3FL restored total levels of GLI3FL and GLI3R in *Ift88*^*fl/fl*^*;Wnt1-Cre;Gli3*^*Δ699/+*^ facial prominences to those more similar to wild-type embryos. GAPDH was used as the loading control. Scale bars in C-E = 500 μm. Inset schematic of facial prominences in F indicate FNP (red) was harvested for the experiment.
(JPG)

S4 Fig *Gli3*^*Δ699/+*^, *Gli3*^*Δ699/∆699*^ and *ΔNGli2;Wnt1-Cre* mutant mice lack facial phenotypes. (A-C) Frontal view of wild-type, *Gli3*^*Δ699/+*^ and *Gli3*^*Δ699/∆699*^ e13.5 embryos. (D) Genotyping results for wild-type, *Gli3*^*Δ699/+*^ and *Gli3*^*Δ699/∆699*^ embryos. (E) Western blot analysis of e11.5 facial prominences from wild-type, *Gli3*^*Δ699/+*^ and *Gli3*^*Δ699/∆699*^ animals. In *Gli3*^*Δ699/∆699*^ mutants, there is no GLI3FL expression.
(JPG)

S5 Fig GLI1 expression is not altered in *Kif3a*^*fl/fl*^*;Wnt1-Cre mutants*. Western blot analysis of wild-type and *Kif3a*^*fl/fl*^*;Wnt1-Cre* embryos for GLI1. Inset schematic of facial prominences indicates FNP, maxillary prominence (MXP) and mandibular prominence (MNP) (red) were harvested for the experiment. GAPDH was used as a loading control.
(JPG)

S6 Fig A minor split in nasal septum was detected in the most ventral aspect in *Gli2*^*fl/fl*^*;Wnt1-Cre* embryos. (A,B) Safranin-O staining of transverse sections through the ventral nasal septum of e15.5 wild-type and *Gli2*^*fl/fl*^*;Wnt1-Cre* embryos. Scale bar = 1000 μm.
(JPG)

S7 Fig Characterization of *ΔNGLI2;Wnt1-Cre* and *Kif3a^fl/fl^; Wnt1-Cre;Δ NGli2* embryos. (A, B) Frontal views of e14.5 wild-type and *ΔNGli2;Wnt1-Cre* embryos. (C, D) Alcian Blue staining on transverse sections through the nasal septum. (E) Western blot analysis of wild-type, *Kif3a*^*fl/fl*^*;Wnt1-Cre* and *Kif3a*^*fl/fl*^*;Wnt1-Cre;ΔNGli2* embryos for GLI2 and MYC. ΔNGLI2 is tagged by MYC. Inset schematic of facial prominences indicates FNP, maxillary prominence (MXP) and mandibular prominence (MNP) (red) were harvested for the experiment.
(JPG)
