# Supplementary figures and images for: Craniofacial Ciliopathies Reveal Specific Requirements for GLI Proteins during Development of the Facial Midline
Source: PLoS Genet. 2016 Nov 1;12(11):e1006351. doi: 10.1371/journal.pgen.1006351 (PMC5089743; doi:10.1371/journal.pgen.1006351)

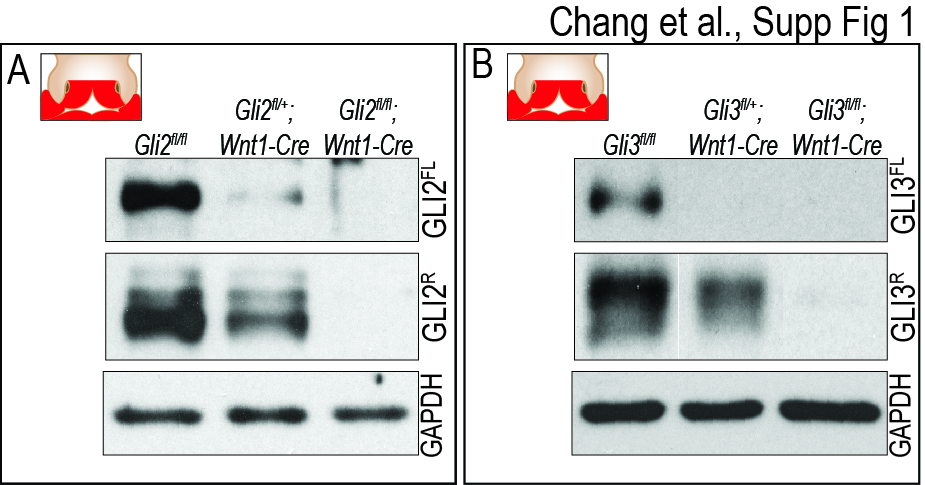

Supplement: S1 Fig — (A) Western blotting analysis performed with protein extract from all facial prominences of wild-type, Gli2fl/+;Wnt1-Cre and Gli2fl/fl;Wnt1-Cre embryos. Both GLI2FL and GLI2R were lost or significantly reduced in Gli2fl/fl;Wnt1-Cre mutant, indicating that the Gli2 gene was efficiently knocked out by Cre activity. (B) Western blotting analysis with all facial prominences of wild-type, Gli3fl/+;Wnt1-Cre and Gli3fl/fl;Wnt1-Cre embryos. GLI3 protein was also largely eliminated in Gli3fl/fl;Wnt1-Cre mutant. Inset schematics of facial prominences in A and B indicates FNP, maxillary prominence (MXP) and mandibular prominence (MNP) (red) were harvested for the experiment. (JPG) [file pgen.1006351.s001.jpg]

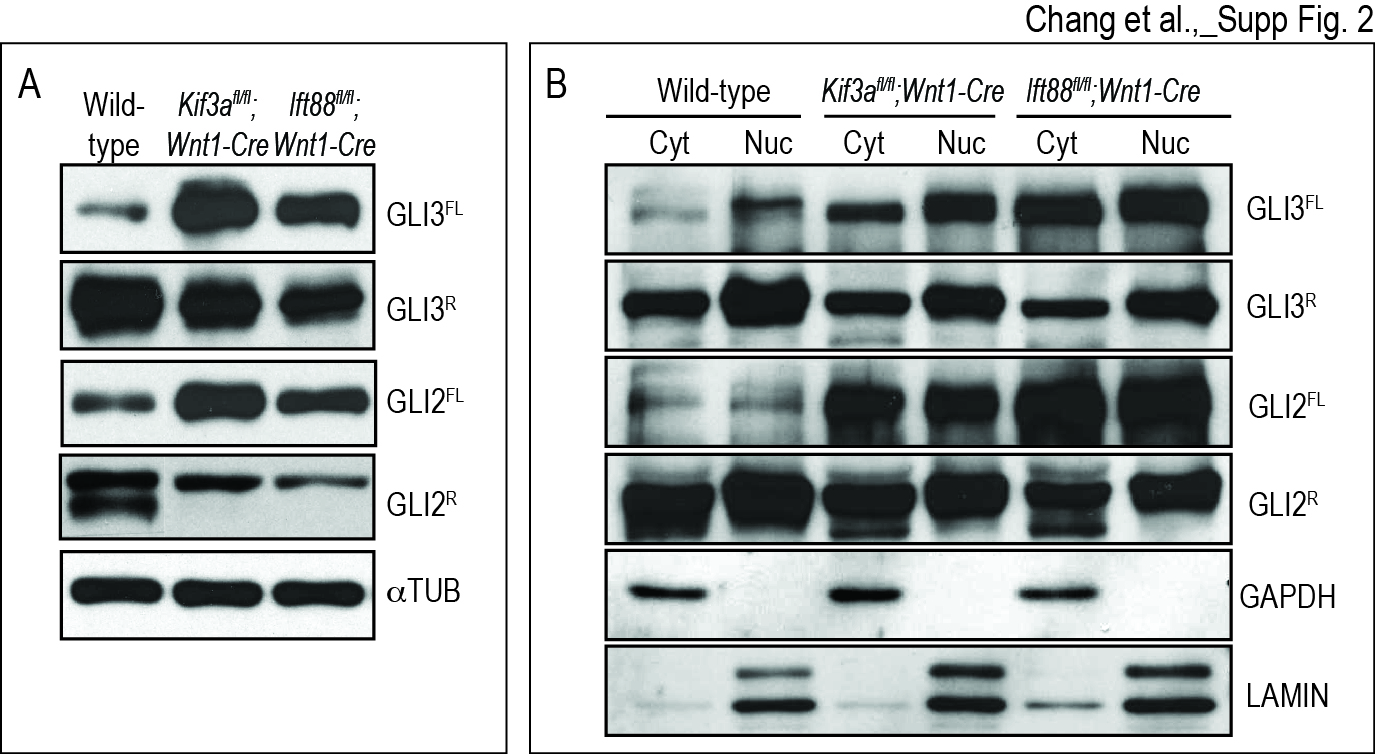

Supplement: S2 Fig — (A) Western blot and (B) nuclear fractionation analysis of GLI3FL, GLI3R, GLI2FL and GLI2R from the FNP of e11.5 embryos with a 2min exposure time. Longer exposure reveals presence of isoforms not observed at shorter exposure time (Fig 2; 30sec). (JPG) [file pgen.1006351.s002.jpg]

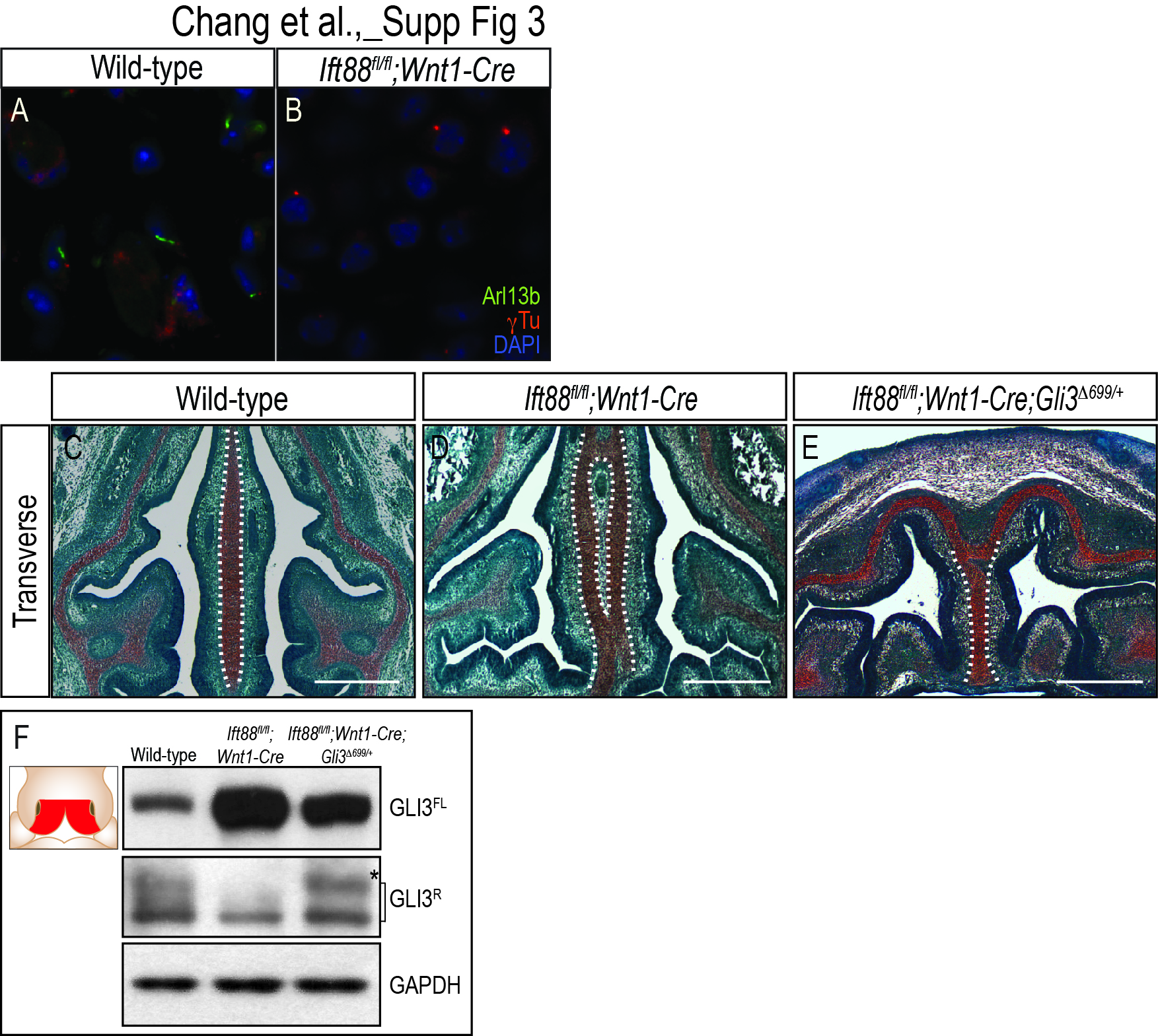

Supplement: S3 Fig — (A, B) Immunostaining for the axonemal marker Arl13b (green) and basal body marker γTubulin (red). (A) The axoneme and basal body are present in mesenchyme of the FNP of wild-type embryos. (B) The axoneme is lost in the mesenchyme of the FNP of Ift88fl/fl;Wnt1-Cre embryos, but γTubulin staining remains, similar to observations in Kif3afl/fl;Wnt1-Cre embryos. Safranin-O staining of transverse sections from e15.5 (C) wild-type, (D) Ift88fl/fl;Wnt1-Cre and (E) Ift88fl/fl;Wnt1-Cre;Gli3Δ699/+ heads. The duplicated nasal septum of the Ift88fl/fl;Wnt1-Cre was restored to a singular cartilaginous element in Ift88fl/fl;Wnt1-Cre;Gli3Δ699/+ (compare D and E; dotted white lines). (F) Western Blot for GLI3 isoforms. Expression of GLI3Δ699R (asterisk) and reduced expression of GLI3FL restored total levels of GLI3FL and GLI3R in Ift88fl/fl;Wnt1-Cre;Gli3Δ699/+ facial prominences to those more similar to wild-type embryos. GAPDH was used as the loading control. Scale bars in C-E = 500 μm. Inset schematic of facial prominences in F indicate FNP (red) was harvested for the experiment. (JPG) [file pgen.1006351.s003.jpg]

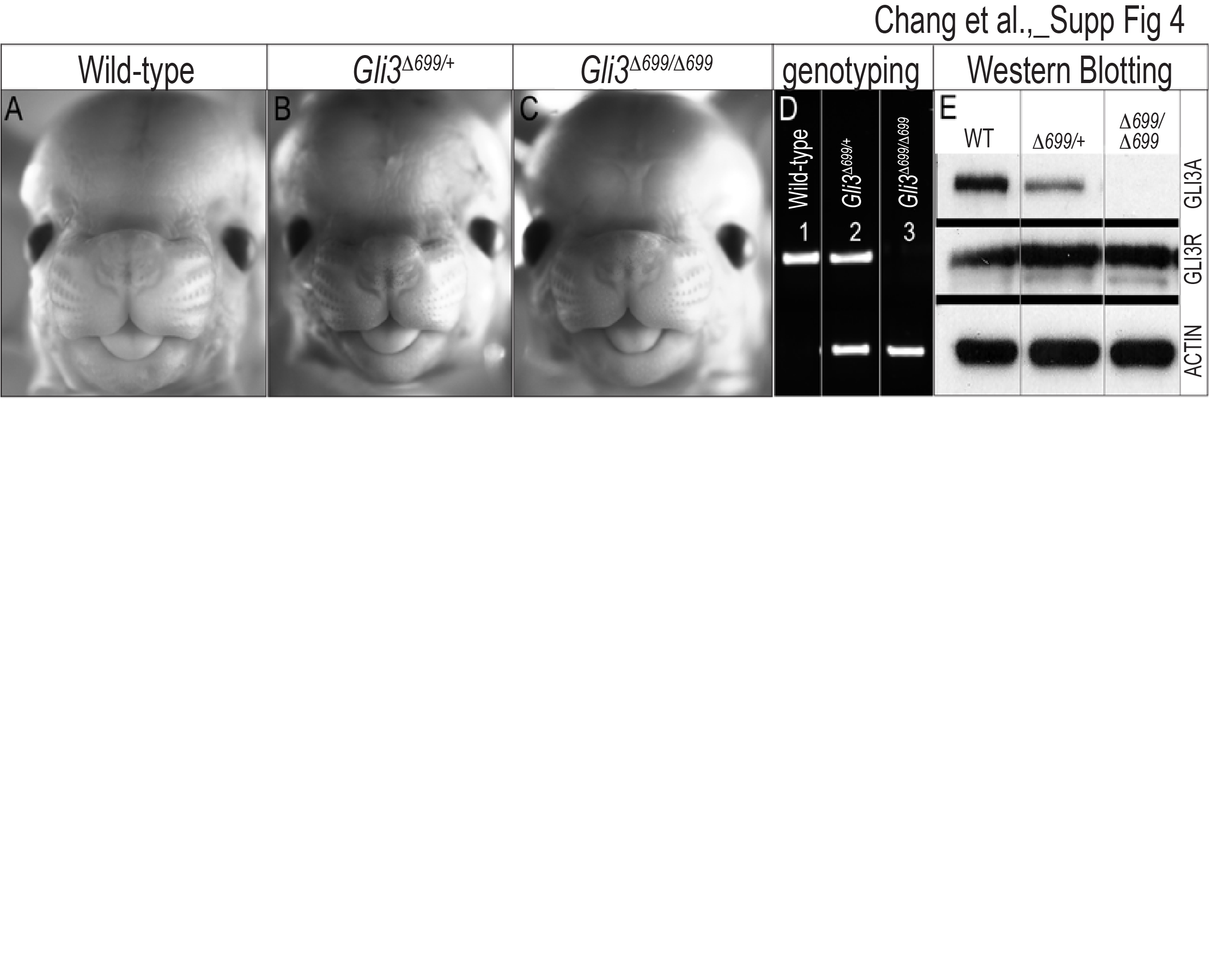

Supplement: S4 Fig — (A-C) Frontal view of wild-type, Gli3Δ699/+ and Gli3Δ699/∆699 e13.5 embryos. (D) Genotyping results for wild-type, Gli3Δ699/+ and Gli3Δ699/∆699 embryos. (E) Western blot analysis of e11.5 facial prominences from wild-type, Gli3Δ699/+ and Gli3Δ699/∆699 animals. In Gli3Δ699/∆699 mutants, there is no GLI3FL expression. (JPG) [file pgen.1006351.s004.jpg]

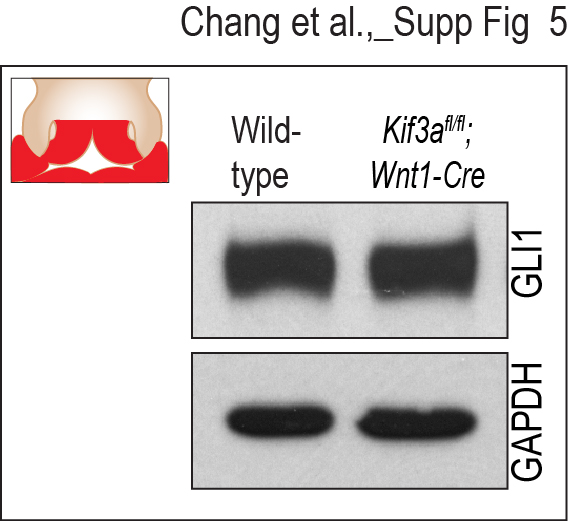

Supplement: S5 Fig — Western blot analysis of wild-type and Kif3afl/fl;Wnt1-Cre embryos for GLI1. Inset schematic of facial prominences indicates FNP, maxillary prominence (MXP) and mandibular prominence (MNP) (red) were harvested for the experiment. GAPDH was used as a loading control. (JPG) [file pgen.1006351.s005.jpg]

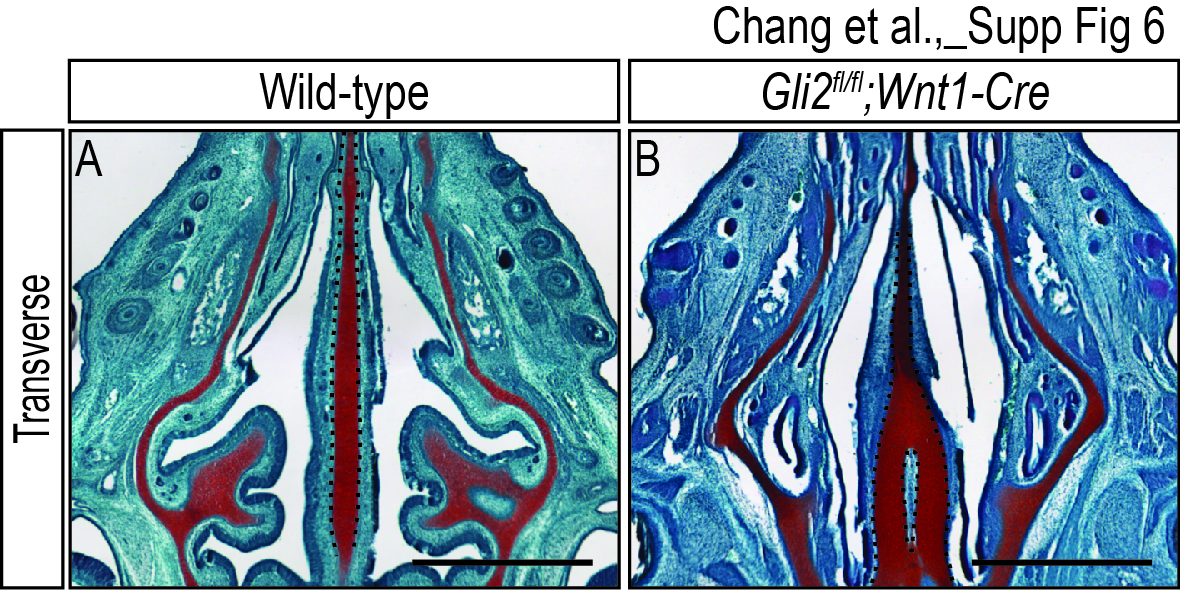

Supplement: S6 Fig — (A,B) Safranin-O staining of transverse sections through the ventral nasal septum of e15.5 wild-type and Gli2fl/fl;Wnt1-Cre embryos. Scale bar = 1000 μm. (JPG) [file pgen.1006351.s006.jpg]

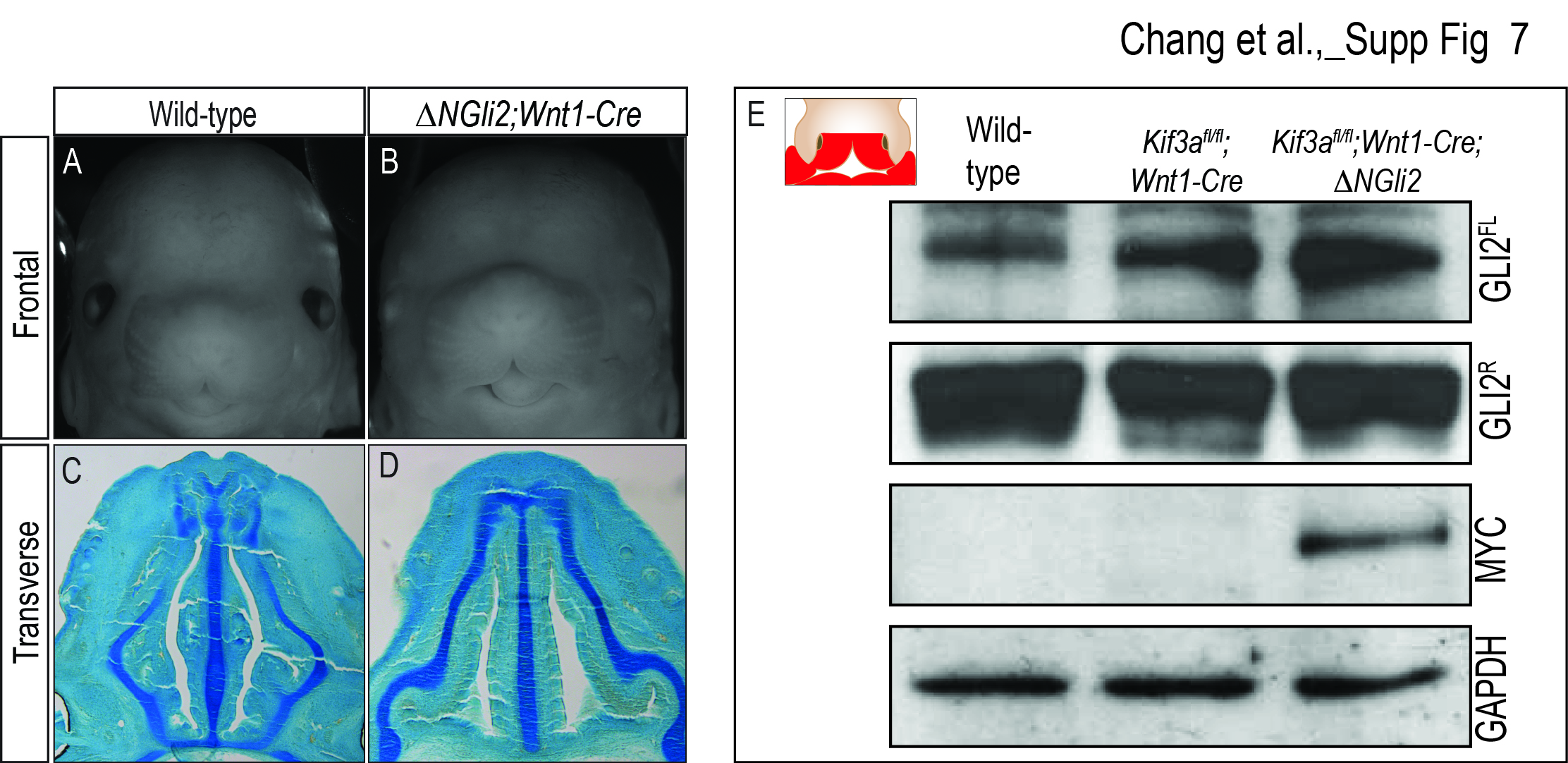

Supplement: S7 Fig — (A, B) Frontal views of e14.5 wild-type and ΔNGli2;Wnt1-Cre embryos. (C, D) Alcian Blue staining on transverse sections through the nasal septum. (E) Western blot analysis of wild-type, Kif3afl/fl;Wnt1-Cre and Kif3afl/fl;Wnt1-Cre;ΔNGli2 embryos for GLI2 and MYC. ΔNGLI2 is tagged by MYC. Inset schematic of facial prominences indicates FNP, maxillary prominence (MXP) and mandibular prominence (MNP) (red) were harvested for the experiment. (JPG) [file pgen.1006351.s007.jpg]
